# Maternal health care seeking behavior: the case of *Haor* (wetland) in Bangladesh

**DOI:** 10.1186/s12889-016-3296-2

**Published:** 2016-07-18

**Authors:** Md. Aminul Haque, Surjya Kanta Dash, Muhammad Abdul Baker Chowdhury

**Affiliations:** Department of Population Sciences, University of Dhaka, Dhaka, 1000 Bangladesh; Department of Biostatistics, Robert Stempel College of Public Health & Social Work, Florida International University, Miami, FL 33199 USA

**Keywords:** Maternal health care (MHC), Antenatal care (ANC) services, Postnatal care (PNC), Hoar areas, Bangladesh

## Abstract

**Background:**

The state of maternal healthcare (MHC) in Bangladesh is a grave concern especially in the remote *haor* areas. In this study, we aimed to determine the factors affecting the utilization of MHC services in the *haor* areas, to discover mothers’ knowledge of MHC, and explore their attitudes toward MHC as well as practices in seeking MHC services.

**Method:**

In this cross-sectional survey (*n* = 400), we randomly selected mothers (aged 15–49 years) from *haor* areas of the Habiganj district of Bangladesh. The study participants’ socio demographic information as well as the extent of their knowledge about MHC, their attitudes, and practices in seeking MHC services were ascertained. The degree of association between the respondents’ socio-demographic characteristics and their health-seeking behavior (before, during, and after childbirth) was assessed by the odds ratio (OR) with 95 % confidence intervals (CI) estimated from the bivariate and multivariable logistic regression analyses.

**Results:**

The mean age of the study participants was 27.26 years. Respondents had an average of 2.64 children, and 88.6 % had at best a primary education or less. Among the study participants, 61 % of mothers had no knowledge about the availability of MHC in the study area, and only 36 % received any antenatal care (ANC). Also, 47 % sought ANC from government healthcare institutions. Irrespective of complications and potential danger signs, 95 % of births were delivered at home with the assistance of untrained birth attendants. Only 19.75 % of mothers and 12.3 % of infants received postnatal care (PNC). Moreover, mothers who had a secondary or tertiary education level had a higher likelihood of receiving ANC (OR: 3.48, 95 % C.I: 1.49–7.63) compared to mothers with no education. Also, mothers aged 25 years or older were less likely (OR: 0.24, 95 % C.I: 0.06–0.095) to give birth in a health facility than mothers who were younger than 25. The low utilization of MHC services can be attributed to many factors such as a lack of communication, a lack of knowledge about MHC services, low income, decision making, and the lack of a companion with whom to visit health services.

**Conclusion:**

To improve MHC utilization, to reach national targets and to save the lives of mothers and newborns, boat or ship-based special healthcare and educational programs should be implemented in the *haor* areas.

## Background

Maternal mortality ratio (MMR) of a country is an important indicator of the overall health status of its mothers. Similar to other developing countries, in Bangladesh, high MMR epitomizes the end point in a lifetime experience in which women encounter gender discrimination, societal neglect and deprivation. Also, high MMR signifies the weakness of the health system to provide effective services and care for the population. The target of the fifth Millennium Development Goal (MDG-5) for Bangladesh was to reduce MMR by 75 % between 1990 and 2015 (i.e. to reduce the MMR to 143 deaths per 100,000 live births). Bangladesh has experienced a gradual decline in its MMR over the past decades, from 500 in 1990 to 194 in 2010 [1]. However, the ratio remains unacceptably high [2, 3]. The government is committed to improving the maternal health situation in the country by adopting special strategies such as the Safe Motherhood Promotion Project (SMPP) [4]. However, the situation remains critical due to inadequate access to healthcare and poor utilization of modern health services. Despite the government’s serious commitment to deliver health facilities to people’s doorsteps through innovative approaches, such as the Essential Service Package (ESP), the utilization of health services is still far below any acceptable standard. Bangladesh did not meet the MDG-5 by the target year, 2015.

Wide disparities exist in the utilization of MHC services between different geographic regions in Bangladesh. According to the 2010 Bangladesh Maternal Mortality Survey Report, the MMR in urban and rural areas was 178 and 198, respectively, with a national average of 194 [5]. These numbers are quite indicative because Bangladesh is essentially an agrarian country with two-thirds of its total population living in rural areas, however, this is one of the highest MMR indicators in the world [6]. Furthermore, approximately 75 % of the babies born to these rural women also die within the first week of their lives [7]. Because modern health services are not equally accessible throughout the diverse geographic areas in the country (e.g. plain, hilly, forest, marshy, or coastal areas), the regional variation in the MMR is striking. For example, in 2010, the MMR in the marshland-dominated northeastern Sylhet Division, was nearly seven times higher (425) than that in the southwestern Khulna Division (64) [5]. The Sylhet division consists of a large number of *haors* which are huge bowl-shaped tectonic depressions that receive surface runoff water during the monsoon. Typically, the low-lying plain land in a *haor* area remains submerged under water for more than six months a year, and during this period, these areas remains completely unreachable. A large area in the eastern part of Bangladesh has been classified as *haor.* Excessive precipitation, flood, and storms in these areas severely affects human life and movement.

### Maternal mortality

Addressing maternal mortality, i.e. the death of a woman during pregnancy or within the first 42 days of post-partum period due to causes directly or indirectly associated with the pregnancy, has been a priority for the global health and development community since the Nairobi Safe Motherhood Conference in 1987 [8]. This conference was followed by numerous international forums at which safe motherhood has always been on the agenda. As a consequence, in 2000 the Millennium Summit of the United Nations set the improvement of maternal health as one of the eight Millennium Development Goals (MDGs) [9]. Accordingly, the MMR, a significant indicator of the overall health status of women in a country, has now become an essential development indicator around the world.

Over the decades, Bangladesh has made some progress in improving maternal and child health. For example, MMR dropped from 570 per 100,000 live births in 1990–91 to 194 per 100,000 live births in 2010 [5, 10]. Similarly, ANC coverage (at least one visit) increased from 27.5 % in 1993–94 to 58.7 % in 2012–13 [11, 12]. But this progress was not enough to achieve the MDG-5 targets in 2015. The Bangladesh government was committed to achieving the Millennium Development Goal (MDG) for maternal mortality through reducing ‘the MMR by three-quarters by the year 2015’ from 1990 levels [10, 13] and is now preparing to address the Sustainable Development Goals 3.1 (SDGs). To lower the national MMR, there is an urgent need to develop effective and affordable programs that ensure proper utilization of MHC services for every woman in the country, especially in the rural areas. Attaining this ambitious goal requires the strengthening of preventive interventions at the community level, ensuring high-quality basic and comprehensive obstetric care, and promoting timely care-seeking from these facilities for maternal emergencies [14].

Care seeking is in many ways the cornerstone of maternal mortality reduction efforts, yet research into how to best promote care seeking in different settings is lacking [15, 16]. The concept of ‘care seeking’ has often been defined in narrow terms in maternal health, with ‘care’ denoting services provided by professionals with appropriate lifesaving skills, and ‘seeking’ denoting transfer of the woman from the home to a health facility [17]. In a recent national survey on maternal health in Bangladesh, the majority of women reported complications during pregnancy and childbirth, but few reported that they sought care from medically trained providers in health facilities, even when they perceived the complication to be life threatening. Most women reported that they accessed MHC in the home or sites other than designated health centers and facilities [18]. Despite the best efforts of the government to deliver healthcare services to people, the situation of pregnancy and childbirth related morbidity and mortality is worse in Bangladesh because of low utilization of maternal health services in remote areas [19].

### MHC delivery system in Bangladesh

The largest part of the country’s health infrastructure and health service system is established under the government’s management and supervision. The Ministry of Health and Family Welfare (MOHFW) is responsible for comprehensive health policy formulation, planning and decision making in Bangladesh. There are two implementation wings under MOHFW: (i) Directorate General of Health Services (DGHS) and (ii) Directorate General of Family Planning (DGFP). The DGHS and DGFP are responsible for implementing all health programs and family planning programs, respectively. Despite having a large population living in a small area, Bangladesh’s public health system is quite well organized. The health service delivery system in the public sector is divided into primary, secondary and tertiary levels. The first contact rural populations have with public health services is in their homes. As the administrative hierarchy rises, the level and sophistication of health services also increase.

A number of studies have been conducted into the difference between MHC utilization in urban and rural areas, but no study has focused exclusively on MHC in the *haor* areas [17, 20–23]. Because of the adverse natural and geographical characteristics of the *haor* region, the community has to adopt differentiated healthcare seeking approaches. This study can provide the government and/or nongovernment service providers with detailed information about formulating effective strategies to attain the SDG by reducing MMR. In this context, the study aimed to determine mothers’ knowledge about MHC services, the pattern of MHC seeking behavior, and factors affecting the utilization of MHC services in the *haor* area.

## Methods

### Study sample

We conducted a cross-sectional study among 400 women age 15–49 years living in two Unions^1^ of the north-eastern Hobigonj District of Sylhet division of Bangladesh who had had at least one live birth in the 5 years preceding the study. The study area and the study participants were selected through a multi-stage random sample procedure. In the first stage, Habiganj district was selected randomly from the six districts of Sylhet Division where *haor*s are located. In the second stage, Ajmirignaj Upazila (a third level government administrative unit) was selected from the Habiganj district as this unit only contains *haor* area. In the third stage, two Unions (local level government administrative units) Ajmiriganj Sadar and Shibpasha were selected randomly from the five Unions of Ajmirignaj Upazila. The list of the total number of married couples having at least one child was prepared with the help of Family Welfare Assistants (FWAs) working in the Unions. The total number of married couples listed in Ajmiriganj Sadarunion and Shibpasha Union was 3240 and 3143, respectively. Then, from each union, 200 married women aged 15–49 years who had experienced at least one pregnancy in the preceding 5 years of the study were randomly selected. The sample size was calculated using the standard formula, assuming the total population size being greater than 10,000. The proportion of married women aged between 15 and 49 with respect to the total female population was 0.34 (*p* = 0.34), so q = 0.66. We set a standard normal deviation at 1.96 corresponding to a 95 % confidence interval and a design effect of 1.0. Using the distribution of the population, the required representative sample size was 345. A total of 400 women (200 from each Union) were interviewed using a semi-structured questionnaire.

### Data collection

Four female research assistants and one supervisor were recruited and trained at the Department of Population Sciences, University of Dhaka, Bangladesh. These four research assistants were involved in the process of the development of the data collection tools, so that they could understand the rationale and theme of each concept and the purpose of the study. All research assistants received training on rapport building, ensuring privacy, confidentiality, and social and cultural sensitivity during data collection. One supervisor was recruited for each Union to lead the data collection process and to resolve any issues related to the data collection. The research assistants described the study purpose and procedures to the study participants, asked for oral consent and enrolled them into the study. The research assistants conducted face-to-face interviews for approximately 1 h with standardized pre tested questionnaires and obtained information on socioeconomic and demographic characteristics, their knowledge of MHC services availability, service delivery schedules, payments mode, ANC, delivery and PNC. The main field work for data collection started on January 01, 2009 and ended on February 15, 2009. The supervisor was present in the field full-time to monitor and ensure the quality of the data collection. All of the information was de-identified before the analyses.

### Data analysis

We checked variables for accuracy and calculated descriptive statistics for age groups, number of children, type of family, husbands’ occupation, household monthly income and education level of participants and their husbands. A chi-square test was performed for ANC and PNC by demographic, socioeconomic, and different types of service providers. We employed binary logistic regression to assess the correlates ANC, place of delivery and assistance at delivery with mother’s age at last birth, birth order, mother’s education, husband’s education, monthly family income, and husband’s occupation. Odds ratios (OR) with 95 % confidence intervals (CI) were estimated. Model adequacy was checked using the Chi-square value of the Hosmer Lomshow test. We also examined the value of 2 Log Likelihood ratio test, AIC, and the area under the receiver operating characteristic (ROC) curve. Data was analyzed using the Statistical Package for the Social Sciences (Version SPSS-12.0 and SPSS-15.0), and considered two-sided inference tests with an alpha <0.05 as statistically significant.

## Results

The distribution of the socio-demographic characteristics of the respondents is presented in Table 1. The mean age of the respondents was 27.62 years. Approximately 37.8 % had no formal education and 50.8 % of the respondents had between 1 and 5 years of schooling. Around 30 % of respondents’ husbands were involved in agricultural activities and 34.3 % were day laborers. The mean household income of the respondents was BDT4339.25 per month (1US$ 1 = BDT 79.5 in 2015) for a mean household size of 6.42 people.

**Table 1:** Distribution of the respondents by their socio-demographic characteristics

Categories		Frequency	Percent	
15–19		33	8.3	
20–24		100	25.0	
25–29		133	33.3	Mean age : 27.62 Years;
30–34		84	21.0	
35–39		45	11.3	
40+		5	1.3	
Total		400	100.0	
Number of children				
1		87	21.8	
2		101	25.3	Mean number of children:2.64/ household
3		118	29.5	
4		60	15.0	
5		31	7.8	
6+		3	0.8	
Total		400	100.0	
Type of family				
Nuclear family		84	21.0	
Extended family		316	79.0	
Total		400	400	
Number of family member				
Less than 5		106	26.5	
6–8		265	66.3	Mean: 6.42
9+		29	7.3	
Total		400	100.0	
Occupation of the husband of the respondents				
Agriculture		118	29.5	
Day Laborer		137	34.3	
Small business		120	30.0	
Service		18	4.5	
Other		7	1.8	
Total		400	100	
Monthly income (TK)				
2000–4000		256	64.0	
4000–6000		93	23.3	Mean: 4339.25
6000–8000		36	9.0	
8000–10,000		7	1.8	
10,000–12,000		8	2.0	
Total		400	100.0	
Level of education	Respondent		Husband of respondent	
	Frequency	Percent	Frequency	Percent
No education	151	37.8	135	33.8
Primary (1–5)	203	50.8	219	54.8
Secondary (6–10)	39	9.8	34	8.5
Higher Secondary (11–12)	7	1.8	5	1.3
Graduation	–	–	7	1.8
Total	400	100.0	400	100.0

It was observed that only 36 % of women who gave birth in the 5 years preceding the survey received at least one instance of ANC from any source. Also, among the women who received ANC, 47.9 % of them sought it from government healthcare institutions i.e. Upazila Health Complex or Union Family Welfare Centre (Table 2). It was observed from the study that only 13.8 % of women who experienced birth in the 5 years preceding the survey received PNC following their last birth. Findings show that only 12.3 % of infants received PNC (Table 2).

**Table 2:** Distribution of respondents by Antenatal Care Visits, sources of ANC, providers and place of delivery

Characteristics	Frequency	Percent
Antenatal care visits at the time of last pregnancy		
Yes	144	36.0
No	256	64.0
Total	400	100
Source of antenatal care		
Government health care institutions	69	47.9
Pharmacy	28	19.4
Quack	47	32.6
Total	144	100.0
Place of delivery		
Home	380	95.0
Health facility	20	5.0
Total	400	100.0
Post-natal care for mothers (*n* = 400)		
Yes	55	13.8
Post-natal care for child (*n* = 400)		
Yes	49	12.3

The three main reasons for receiving ANC were headache (21 %), abdominal pain (32.7 %), and excessive vomiting (27. 2 %). The main reasons for not seeking ANC in a facility were high cost (24.8 %), lack of money (26.8 %), remote location (7.4 %) and bad transportation (20.0 %) (Table 3). The study findings showed 95 % of deliveries occurred in the home, and only 5 % (20) of deliveries occurred at health centers (Table 3).

**Table 3:** Distribution of respondents by problems for which they did and did not sought ANC

Problems	Response		Percent of cases
	N	Percent	
Reason for seeking ANC			
Regular check-up	10	3.7	6.8
Headache	57	21.0	38.8
High blood pressure	11	4.0	7.5
Tetanus	9	3.3	6.1
Abdominal pain	89	32.7	60.5
Injury	17	6.3	11.6
Excess vomiting	74	27.2	50.3
Other	5	1.8	3.4
Total	272	100.0	185.0
Reason for not seeking ANC			
Lack of need of ANC services	66	10	26.1
Expensive	164	24.8	64.8
Lack of money	177	26.8	70.0
Too far/distance	48	7.3	19.0
Problem with transportation	125	18.9	49.4
Lack of companion	10	1.5	4.0
No permission from family	70	10.6	27.7
Total	660	100.0	260.9

^a^ *Multiple responses*

The bivariate analysis shows that receiving ANC was significantly associated with the socio-economic and demographic variables like mother’s age at birth, birth order, mother’s education, husband’s education and occupation, and household family income (Table 4). Moreover, mother’s age at birth, birth order, and mother’s education was significantly associated with type of ANC provider (Table 5).

**Table 4:** Socio-demographic profile by antenatal care received

Characteristics	Antenatal care received				Total (*N*)	*p-values*
	Yes		No			
	n	%	n	%		
Mothers age at birth						
Less than 25	66	49.6	67	50.4	133	
25–29	45	33.8	88	66.2	133	0.000
30–34	27	32.1	57	67.9	84	
More than 35	6	12.0	44	88.0	50	
Birth Order						
1	48	55.2	39	44.8	87	
2	36	33.6	65	64.4	101	0.000
3	41	34.7	77	65.3	118	
4	19	20.2	75	79.8	94	
Type of family						
Nuclear family	13	35.1	24	64.9	37	
Extended family	131	36.1	232	63.9	363	0.908
Mother’s education						
No education	38	25.2	113	74.8	151	
Primary	71	35.4	132	65.0	203	0.000
Secondary and more	35	76.1	11	23.9	46	
Husband’s education						
No education	32	23.7	103	76.3	135	
Primary	81	37.0	138	63.0	219	0.001
Secondary and more	31	67.4	15	32.6	46	
Husband’s occupation						
Agriculture	40	33.9	78	66.1	118	
Laborer	31	22.6	106	77.4	137	
Small business	55	45.8	65	54.2	120	0.000
Service	14	77.8	4	22.2	18	
Others	4	57.1	3	42.9	7	
Family income						
2000–4000	81	31.6	175	68.4	256	
4000–6000	36	38.7	57	61.3	93	0.000
6000–8000	18	50.0	18	50.0	36	
More than 8000	9	60.0	6	40.0	15

**Table 5:** Background characteristics of participants who received ANC by different type of provider

Background characteristics	Medically trained providers				Non-trained provider		Total (*N*)	*p- values*
	MBBS doctor		Nurse/paramedic					
	N	%	N	%	N	%		
Mother’s age at birth								
Young aged mother (≤25 years)	23	34.8	29	43.9	14	21.2	66	0.022
Old aged mother (>25 years)	17	21.8	28	35.9	33	42.3	78	
Birth order								
1	21	43.8	18	37.5	9	18.8	48	
2	6	16.7	18	50.0	12	33.3	36	0.038
3	9	22.0	15	36.6	17	41.5	41	
4	4	21.1	6	31.6	9	47.4	19	
Type of family								
Nuclear family	4	30.8	7	53.8	2	15.4	13	
Extended family	36	27.5	50	38.2	45	34.4	131	0.353
Mother’s education								
No education	5	13.2	17	44.7	16	42.1	38	
Primary	17	23.9	29	40.8	25	35.2	71	0.005
Secondary and more	18	51.4	11	31.4	6	24.3	35	
Husband’s education								
No education	6	18.8	10	31.3	16	50.0	32	
Primary	23	28.4	33	40.7	25	30.9	81	0.128
Secondary and more	11	35.5	14	45.2	6	19.4	31	

*p-values* obtained from Chi-Squire test

The analysis also shows that, mother’s age at birth, type of family, and birth order were significantly associated with determining place of delivery, as well as the type of assistance during delivery (Table 6). Younger mothers, educated mothers, and mothers with husbands with higher a level of education received more PNC (Table 7). The majority of the respondents (85 %) opined that a PNC check-up was not needed.

**Table 6:** Place of delivery according to background characteristics

Background characteristics	Home		THC/ UFWC		Total (*N*)	*P*-Value
	N	%	N	%		
Mother’s age at birth						0.000
Young aged mother (≤25)	117	88.0	16	12.0	133	
Old aged mother (>25)	263	98.5	4	1.5	267	
Birth Order						0.000
1	74	85.1	13	14.9	87	
2	97	96.0	4	4.0	101	
3	117	99.2	1	0.8	118	
4	92	97.9	2	2.1	94	
Type of family						0.001
Nuclear family	31	83.8	6	16.2	37	
Extended family	349	96.1	14	3.9	363	
Mother’s education						0.111
No education	143	94.7	8	5.3	151	
Primary	196	96.6	7	3.4	203	
Secondary and more	41	89.1	5	10.9	46	
Husband’s education						0.301
No education	127	94.1	8	5.9	135	
Primary	211	96.3	8	3.7	219	
Secondary and more	42	91.3	4	8.7	46	

*p-values* obtained from Chi-Squire test

**Table 7:** Percentage of the respondents received postnatal care by some socio-economic and demographic factor

Background characteristics	Postnatal care received				Total	*p*- value
	Yes		No			
	N	%	N	%	N	
Mother’s age at birth						
Young aged mother (≤25)	29	21.8	104	78.2	133	0.506
Old aged mother (>25)	50	18.7	217	81.3	267	
Mother’s education						
No education	22	14.6	129	85.4	151	
Primary	46	22.7	157	77.3	203	0.126
Secondary and more	11	23.9	35	76.1	46	
Husband’s education						
No education	20	14.8	115	85.2	135	
Primary	49	22.4	170	77.6	219	0.208
Secondary and more	10	21.7	36	78.3	46	

*p-values* obtained from Chi-Squire test

Table 8 shows the results from the logistic regression analysis with 95 % confidence interval for the use of ANC. Only mothers’ education level was found to be a significant predictor of receiving ANC adjusted by other covariates. Mothers who have primary education were 3.38 (95 % CI: 1.39, 8.70) times more likely to receive ANC compared to the mothers without education. Similarly, mothers with secondary education had higher odds (OR: 3.48, 95 % CI: 1:50, 7.63) of receiving ANC compared to the mothers without education. No association was observed for mother’s age at birth, birth order, husband’s education, family income and/ or husband’s occupation.

**Table 8:** Logistic regression estimates for use of antenatal care

Independent variable	Regression coefficient	Odds ratio = Exp (B)	95 % C.I. for Exp (B)	
			Lower	Upper
Mother’s age at last birth				
Less than 25 years (RC)		1.00		
25 years and more	−0.390	0.677	.372	1.232
Birth order				
First birth (RC)		1.00		
Second birth and more	−0.414	0.661	.333	1.314
Mother’s education				
No education (RC)		1.00		
Primary	1.248	3.378*	1.394	8.696
Secondary and more	1.217	3.482*	1.495	7.631
Husband’s education				
No education (RC)		1.00		
Primary	0.888	2.429	.984	6.001
Secondary and more	0.576	1.779	0.825	3.839
Monthly family income				
Less than Tk. 2500 (RC)		1.000		
TK. 2500 and more	.084	1.087	0.657	1.799
Occupation of husband				
Laborer (RC)		1.000		
Other than laborer	0.488	1.630	.932	2.849
Constant	−1.092	.336		
HosmerLomshow test = Chi-square 7.942 sig. 0.439				
−2 Log likelihood = 466.256				
Nagelekerke R. = .188				

* = *p* <0.05, *RC* reference category

The results also show that only mother’s age was significantly associated with predicting the place of delivery. It was found that, mothers aged 25 years and older were less likely (OR: 0.24, 95 % CI: 0.06, 0.95) to give birth in a healthcare facility than the mothers who are less than 25 years of age (Table 9). Neither birth order nor family type had influence on predicting the place of delivery.

**Table 9:** Logistic regression estimate for place of delivery of respondents

Independent variable	Regression coefficient	Odds ratio = Exp (B)	95 % C.I. for Exp (B)	
			Lower	Upper
Mother’s age at last birth				
Less than 25 years (RC)		1.00		
25 years and more	−1.436	0.24*	0.059	0.954
Birth order				
First birth (RC)		1.00		
Second birth and more	−0.971	0.379	0.115	1.251
Family type				
Nuclear family (RC)		1.00		
Extended family	−0.920	0.399	0.135	1.181
Constant	4.314	74.725		
HosmerLomshow test = Chi-square 5.73, sig. 0.125				
−2 Log likelihood = 133.33				
Nagelekerke R. = .188				

* = *p* <0.05, *RC* reference category

Similar to the place of delivery, only mothers’ level of education was significantly associated with predicting assistance during delivery. It was found that mothers with primary (OR: 0.48, CI: 0.18, 0.95), secondary or tertiary (OR: 0.41, 95 % CI: 0.22, 0.97) were less likely to give birth assisted by a traditional birth attendant compared to mothers with no education. Mothers age at last birth, birth order, and/ or family type (nuclear vs. extended) had no influence on the prediction of assistance at the time of delivery (Table 10).

**Table 10:** Logistic regression estimates for assistance at delivery of respondents

Independent variable	Regression coefficient	Odds ratio = Exp (B)	95 % C.I. for Exp (B)	
			Lower	Upper
Mother’s age at last birth				
Less than 25 years (RC)		1.00		
25 years and more	0.009	1.009	0.475	2.142
Birth order				
First birth (RC)		1.00		
Second birth and more	0.626	1.871	0.832	4.208
Family type				
Nuclear family (RC)		1.00		
Extended family	0.057	1.059	.438	2.558
Mother’s education				
No education (RC)		1.00		
Primary	−.783	0.457*	0.179	0.948
Secondary and more	−0.887	0.412*	0.216	0.968
Constant	−1.024	.359		
HosmerLomshow test = Chi-square 1.72, sig. 0.786				
−2 Log likelihood = 357.33				
Nagelekerke R. = 0.056				

* = *p* <0.05, *RC* reference category

## Discussion

All the survey respondents were rural people of a geographically adverse area, with little formal education whose livelihood is mainly based on agriculture. *Haor*s occupy a large portion of Bangladesh, and this region is typically inaccessible, with the plains being submerged under water for more than 6 months of the year, causing transport and communication facilities to become very poor. The study found that among ANC receivers half of the women seek ANC from government healthcare institutions, i.e. Upazila Health Complex or Union Family Welfare Centre. This percentage is lower than the national average for rural women of Bangladesh who received ANC from government facilities [3]. Receiving ANC services from medically trained personnel is very important for the wellbeing of mothers and new-born children. The number of ANC visits and the timing of the first check-up are both considered important in detecting and preventing an adverse pregnancy outcome. As per the World Health Organization (WHO) standard, every pregnant woman should receive ANC within the first trimester irrespective of any problem faced by them. This study’s survey respondents only seek ANC when they face a particular problem, and the percentage is very low (6.8 %) compared to the national average. This may be due to low level of education, lack of awareness, etc. Moreover, the study also found that a lack of money is the prominent reason for not seeking ANC. This finding is supported by the low economic status of the respondents.

Half of the respondents mentioned transportation problems as a reason for not seeking ANC. In the study area, transportation and communication systems are not well developed, and the means of transportation available for going to the healthcare institutions such as boats and rickshaws are very limited. Within this adverse geographic location, it was observed that mothers who had higher educational attainment were far more likely to use ANC. However, the education level of the mothers’ in the study area was very low compared to the national average [1].

Delivery care is another component of MHC, which is assessed by the place of delivery and assistance during the delivery. The percentage of deliveries at healthcare facilities in the study area was approximately half the national average for rural areas [3]. Deliveries attended by untrained traditional birth attendants were higher than the national average of 62.5 %. Postnatal check-ups provide an opportunity to assess and treat delivery complications and to counsel mothers on how to care for themselves and their newborns. Younger women, educated mothers and mothers who have husbands with higher education levels received more PNC. The percentage of women and infants who received PNC was also much lower than the national average. National statistics show that 29 % of women and 30 % of children received PNC from medically trained providers within 42 days after delivery [3]. A number of factors contribute to not seeking PNC, including lack of money, lack of need of the PNC services, prohibitive cost, transportation problems, etc. The majority of the respondents (85 %) opined that regular check-ups were not needed. This implies that they do not seek any care if they do not face any problem. However, we know from the literature that postnatal visits should be made within two days after giving birth [24]. Bivariate analysis of receiving PNC and different socio-economic and demographic factors revealed that only variables like mother’s age at birth, mother’s education, and husband’s level of education were significantly associated with receiving PNC.

### Limitations

There are several limitations of the present study. While conducting the interviews, we had to depend on the information provided by mothers. Information could, thus, have been subject to recall bias. However, we were careful in collecting the data they provided and in the analysis and interpretation of the results. The findings are only generalizable to the *haor* areas since the studied socio-demographic and economic characteristics are different from the populations in other parts of the country.

## Conclusions

The utilization of MHC in the *haor* area is below the standard level. The status of main indicators of MHC utilization such as receiving ANC from medically trained providers, giving birth at a health facility, delivery assisted by medically trained providers, receiving PNC for mother and new-born baby from medically trained providers, are all below the national average. Only mothers’ education and mothers’ age at last birth influenced the utilization of ANC, birth facilities, and the use of trained providers. In this study, mothers’ age at birth, birth order, mothers’ education, husbands’ education, family type, and family income were found to be associated with MHC utilization. Improved MHC utilization can reduce maternal mortality and maternal morbidity. To achieve the Sustainable Development Goal (SDG) 3.1, MHC utilization must be improved in remote parts of the country. The study, therefore, concludes that the Bangladesh government needs to act deliberately to address the factors responsible for the observed MHC utilization differential in *haor* areas compared to other regions of the country.

## Abbreviations

ANC, antenatal care; DGFP, directorate general of family planning; DGHS, directorate general of health services; ESP, essential service package; GO, government organization; MBBS, bachelor of medicine and bachelor of surgery; MDG, millennium development goals; MHC, maternal health care; MMR, maternal mortality ratio; MOHFW, ministry of health and family welfare; NGO, non-government organization; PNC, post-natal care; SDG, sustainable development goals; SMPP, safe motherhood promotion project

## Footnotes

1 Lowest administrative unit in Bangladesh.
